# Tumor growth rate during re-challenge chemotherapy with previously used agents as salvage treatment for metastatic colorectal cancer: A retrospective study

**DOI:** 10.1371/journal.pone.0257551

**Published:** 2021-09-24

**Authors:** Masashi Ishikawa, Atsuo Takashima, Yusuke Nagata, Ryoichi Sawada, Masahiko Aoki, Hiroshi Imazeki, Hidekazu Hirano, Hirokazu Shoji, Yoshitaka Honma, Satoru Iwasa, Natsuko Okita, Ken Kato, Masayuki Saruta, Narikazu Boku

**Affiliations:** 1 Gastrointestinal Medical Oncology Division, National Cancer Center Hospital, Chuo-ku, Tokyo, Japan; 2 Division of Gastroenterology and Hepatology, Department of Internal Medicine, The Jikei University School of Medicine, Minato-ku, Tokyo, Japan; Centro di Riferimento Oncologico, ITALY

## Abstract

**Background:**

In clinical practice, the same chemotherapeutic agents are occasionally reused (re-challenge) after failure of all available standard chemotherapy options for metastatic colorectal cancer (mCRC). However, the benefits of re-challenge chemotherapy (Re-Cx) are unclear. This retrospective study evaluated the efficacy of Re-Cx, focusing on the tumor growth rate (TGR).

**Methods:**

The study included mCRC patients with measurable lesions who received Re-Cx from November 2011 to October 2018 at National Cancer Center Hospital. Re-Cx was defined as re-administration of agents which had been used in prior lines of chemotherapy and discontinued due to disease progression. We compared the TGR immediately after initiating Re-Cx regimens with that observed at the time of disease progression during prior chemotherapy (Prior-Cx) immediately before Re-Cx.

**Results:**

Of the 25 patients who received Re-Cx, five patients received two Re-Cx regimens. Therefore, a total of 30 cases of Re-Cx were analyzed in this study. The regimens of Re-Cx were oxaliplatin based (19 cases), irinotecan based (8 cases), and others (3 cases). Although the objective response rate to Re-Cx was 0%, the disease control rate was 60% (18 cases), and 40% (12 cases) showed some tumor shrinkage. We compared the effects of Re-Cx and Prior-Cx by the TGR and found that the TGR of Re-Cx was slower than that recorded in Prior-Cx in 26 of 30 cases (87%). In particular, the ratio of% TGR <0, which indicates tumor shrinkage, was obtained in 13 of 30 cases (43.3%). The median progression-free survival and overall survival after Re-Cx were 3.8 and 6.57 months, respectively.

**Conclusion:**

We found that Re-Cx may have some anti-tumor efficacy as salvage treatment for mCRC and these results also suggested the clinical benefits of Re-Cx.

## Introduction

Colorectal cancer is the third most common type of cancer and the second-leading cause of cancer-related death worldwide. It is estimated that >1.8 million new colorectal cancer cases and 881,000 deaths occurred in 2018, accounting for approximately 10% of all cancer cases and deaths [1].

The key drugs for the treatment of metastatic colorectal cancer (mCRC) are fluorouracil (5-FU), irinotecan, oxaliplatin, and several molecular targeted agents (e.g., bevacizumab, ramucirumab, aflibercept, cetuximab, and panitumumab) [2–5]. Recently, regorafenib and trifluridine-tipiracil (TAS-102) have been recognized as a standard salvage-line chemotherapy for refractory mCRC [6–10]. These drugs contribute to prolongation of overall survival (OS) in patients with mCRC, reaching a median of 30 months [11, 12]. Cancer has the ability to become resistant to many different types of drugs [13], In clinical practice, we make a strategy to use the available drugs at the optimal time. Usually, after using several drugs, or after using all available drugs, the disease progresses and the patient is no longer in a treatable state. However, there are rare patients who remain in good medical condition and are eager to receive chemotherapy even after treatment with all the available drugs has failed. Chemotherapeutic agents which were discontinued due to disease progression during prior lines of chemotherapy are rarely reused (re-challenge) in these patients because there is no other way. However, the benefits of re-challenge chemotherapy (Re-Cx) are unclear.

The tumor growth rate (TGR) is calculated based on changes in tumor volume and is useful in evaluating the impact of treatment on the kinetics of tumor growth. In recent studies, TGR has been used to assess the efficacy of chemotherapeutic agents and the prognosis of cancer patients [14, 15]. Considering that either regorafenib or TAS-102 does not markedly reduce the size of tumors, but leads to stable disease in many patients [6, 7], a decrease in tumor growth through salvage treatment may positively impact patient outcomes. However, there is a limited number of Re-Cx studies focusing on TGR in mCRC.

This study investigated the efficacy of Re-Cx focusing on the TGR immediately after initiating Re-Cx compared with that observed at the time of disease progression during prior chemotherapy (Prior-Cx) immediately before Re-Cx.

## Methods

### Patients

Re-Cx was defined as re-administration of the same agents which had been previously discontinued due to disease progression in previous lines of chemotherapy. Prior-Cx was defined as the last line of chemotherapy administered immediately prior to Re-Cx.

This is a retrospective study. We selected patients with mCRC who received Re-Cx at our institution from November 2011 to October 2018. The selection criteria were as follows: age ≥20 years, Eastern Cooperative Oncology Group PS of 0–2, presence of a measurable lesion at the initiation of Prior-Cx, failure of 5-FU, irinotecan, oxaliplatin, bevacizumab, and panitumumab/cetuximab for wild-type *RAS*, discontinuation of all these drugs due to progressive disease (PD), 3 (or 4) times computed tomography (CT) examination showing the smallest tumor size and disease progression during Prior-Cx, (immediately before Re-Cx if not the same CT showing disease progression during Prior-Cx), the smallest tumor size during Re-Cx. For patients who received 2 Re-Cx regimens, each regimen was counted as an independent case (1 case / 1 Re-Cx regimen).

### TGR

TGR is used to estimate the kinetics of tumor volume, and %TGR expresses the percentage of change in tumor volume over 1 month according to information obtained from two CT scans before and during treatment with Prior-Cx and Re-Cx. This parameter assesses measurable disease defined by the Response Evaluation Criteria in Solid Tumors (RECIST) version 1.1 [16].

TGR was calculated using the following formula [17–19]: TGR = 3 × Log (Dt / D0) / t; where *t* is the interval time (months) between two CT scans, *D* is the sum of the largest diameters of the measurable disease as per RECIST version 1.1 (new lesions and non-measurable lesions were excluded) which virtually represents one lesion with size D, D0 at baseline and Dt at the time point of t after treatment. *R* represents the radius of the sphere with the size of D, and the tumor volume (V) is approximated using the formula V = 4/3 × π × R^3^. Assuming that tumor growth is exponential, the tumor volume at time t (Vt) can be calculated using the formula: Vt = V0 exp(TGR × t), where *V0* is the volume at baseline, *TGR* is the tumor growth rate, and *exp(TGR)* represents the exponential of TGR. Finally, %TGR is obtained by the following transformation of the formula: %TGR = 100 × [exp(TGR) − 1].

### Assessment

This retrospective study assessed the efficacy of Re-Cx as salvage chemotherapy for mCRC in terms of TGR, overall response rate (ORR), disease control rate (DCR), progression-free survival (PFS), and OS. Objective tumor response was assessed according to the RECIST version 1.1.

### Statistical analysis

OS was defined as the time from the initiation of Re-Cx to the date of death due to any cause, or censored at the last contact for surviving patients. PFS was defined as the date from the initiation of Re-Cx to the date of disease progression or death, or censored at the last follow-up for surviving patients without disease progression. OS and PFS curves were estimated using the Kaplan–Meier method. The median follow-up time for survival was calculated using the reverse Kaplan–Meier method. All *p* values were two-sided and values < 0.05 denoted statistical significance. Statistical analyses were performed using the EZR software for Windows version 1.37. This study was approved by the ethics committee of the National Cancer Center Hospital.

## Results

### Patients

The CONSORT diagram is shown in S1 Fig. There were 46 cases (41 patients) who received Re-Cx. Of those, 16 cases were excluded because of insufficient CT examination (8 cases), absence of measurable lesions (7 cases), and discontinuation of prior-Cx due to allergy (1 case). Finally, as five patients received two Re-Cx regimens, a total of 30 cases (25 patients) were analyzed in this study.

Background information of patients immediately before the initiation of Re-Cx is shown in Table 1. The median age was 62 years. 76% of the patients were male. Most of the patients had an Eastern Cooperative Oncology Group PS of 0 or 1 (96%) and a left-side primary tumor (84%). The *RAS* status was wild and the *BRAF* status was unknown in 76% of the patients. The clinical courses of the initial Re-Cx chemotherapy regimens in the 30 cases are shown in Table 2. Collectively, the response achieved during initial Re-Cx was complete response (CR) in 1 case (3%), partial response in 12 cases (40%), stable disease in 10 cases (33%), PD in 1 case (3%), and non-CR/non-PD in 6 cases (20%). The median duration of treatment was 10.5 months (range: 1.4–46.8 months), and the median interval between initial Re-Cx and Re-Cx was 12.4 months (range: 3.8–54.6 months).

**Table 1:** Background information of the 25 patients at the initiation of Re-Cx.

Median age (years)	62 (41–85)
Sex	
Male	19
Female	6
ECOG PS	
0	5
1	19
2	1
Location	
Right	4
Left	21
Number of metastatic sites	
1	4
2	11
>2	10
RAS status	
Wild type	19
Mutant	5
Unknown	1
BRAF status	
Wild type	6
Mutant	0
Unknown	19
Prior used agent	
Cytotoxic agents	
5-FU, capecitabine, S-1	25
CPT-11	25
OHP	25
TAS-102	19
Anti-VEGF antibody	
BV	24
Ramucirumab	0
Aflibercept	0
Regorafenib	15
Anti-EGFR antibody for wild-type RAS (n = 19)	
Cmab	13
Pmab	11

**Table 2:** Initial Re-Cx (30 cases).

Group	Regimen	Treatment line, median [range]	Best response CR/PR/SD/PD/non-CR non-PD	Treatment duration, median [range] (months)	Interval between initial and Re-Cx, median [range] (months)
OHP base (n = 19)	FOLFOX	1	0 / 1 / 0 / 0 / 1	8.6 [2.5–14.7]	27.4 [26.0–28.7]
	(n = 2)				
	FOLFOX+BV	1 [1–6]	1 / 5 / 2 / 0 / 0	7.1 [2.5–27.9]	15.4 [5.4–54.6]
	(n = 8)				
	CapeOX	1	0 / 0 / 0 / 0 / 1	7.0	13.4
	(n = 1)				
	CapeOX+BV	1 [1–2]	0 / 2 / 2/ 0 / 0	10.4 [8.4–10.9]	9.8 [5.2–26.7]
	(n = 4)				
	SOX+BV	2 [1–2]	0 / 0 / 2 / 1 / 1	3.5 [1.4–14.0]	25.1 [3.8–52.3]
	(n = 4)				
CPT-11 base (n = 10)	CPT-11+Cmab	4 [3–5]	0 / 2 / 1 / 0 / 0	14.7 [6.7–18.1]	6.3 [6.2–7.9]
	(n = 3)				
	FOLFIRI+BV	2 [1–3]	0 / 0 / 2 / 0 / 2	14.2 [13.0–32.2]	15.9 [7.4–25.3]
	(n = 4)				
	FOLFIRI+Cmab	2	0 / 0 / 0 / 0 / 1	30.4	10.6
	(n = 1)				
	FOLFIRI+Pmab	1	0 / 1 / 1 / 0 / 0	25.2 [3.6–46.8]	10.2 [6.7–13.7]
	(n = 2)				
Other (n = 1)	TAS-102	4	0 / 1 / 0 / 0 / 0	11.9	10.4
Total (n = 30)		1 [1–6]	1 / 12 / 10 / 1 / 6	10.5 [1.4–46.8]	12.4 [3.8–54.6]

Prior-Cx agents used in the 30 cases were regorafenib (6 cases), TAS-102 (7 cases), and others (17 cases) (Table 3). In Prior-Cx, the median PFS was 2.2 months (95% confidence interval [CI]: 1.84–2.69), while the ORR and DCR were 0% and 30%, respectively.

**Table 3:** Prior-Cx regimens (30 cases).

Agent	Regimen	Treatment line 3/4/5/6/≥6	Best response CR/PR/SD/PD	%TGR, median [range]	Treatment duration, median [range] (months)
Regorafenib (n = 6)		1 / 2 / 0 / 2 / 1	0 / 0 / 0 / 6	5.89 [1.67–11.04]	1.2 [1.0–2.3]
TAS-102 (n = 7)		1 / 1 / 5 / 0 / 0	0 / 0 / 3 / 4	6.64 [0.51–21.27]	1.3 [0.1–4.6]
Others (n = 17)	FOLFOX+BV (n = 2)				
	FOLFIRI+BV (n = 1)				
	CPT-11+BV (n = 1)				
	CPT-11+Pmab (n = 2)	3 / 2 / 4 / 2 / 6	0 / 0 / 6 / 11	9.92 [0.05–24.84]	1.9 [0.1–9.0]
	CPT-11+Cmab (n = 2)				
	CPT-11 (n = 1)				
	Pmab (n = 1)				
	Clinical trial (n = 7)				
Total (n = 30)		5 / 5 / 9 / 4 / 7	0 / 0 / 9 / 21	7.58 [0.05–24.84]	1.4 [0.1–9.0]

The Re-Cx regimens were oxaliplatin-based (63%), irinotecan-based (27%), or others (10%). Re-Cx was often performed after the sixth-line of therapy (67%) (S1 and S2 Tables).

**Fig 1:**
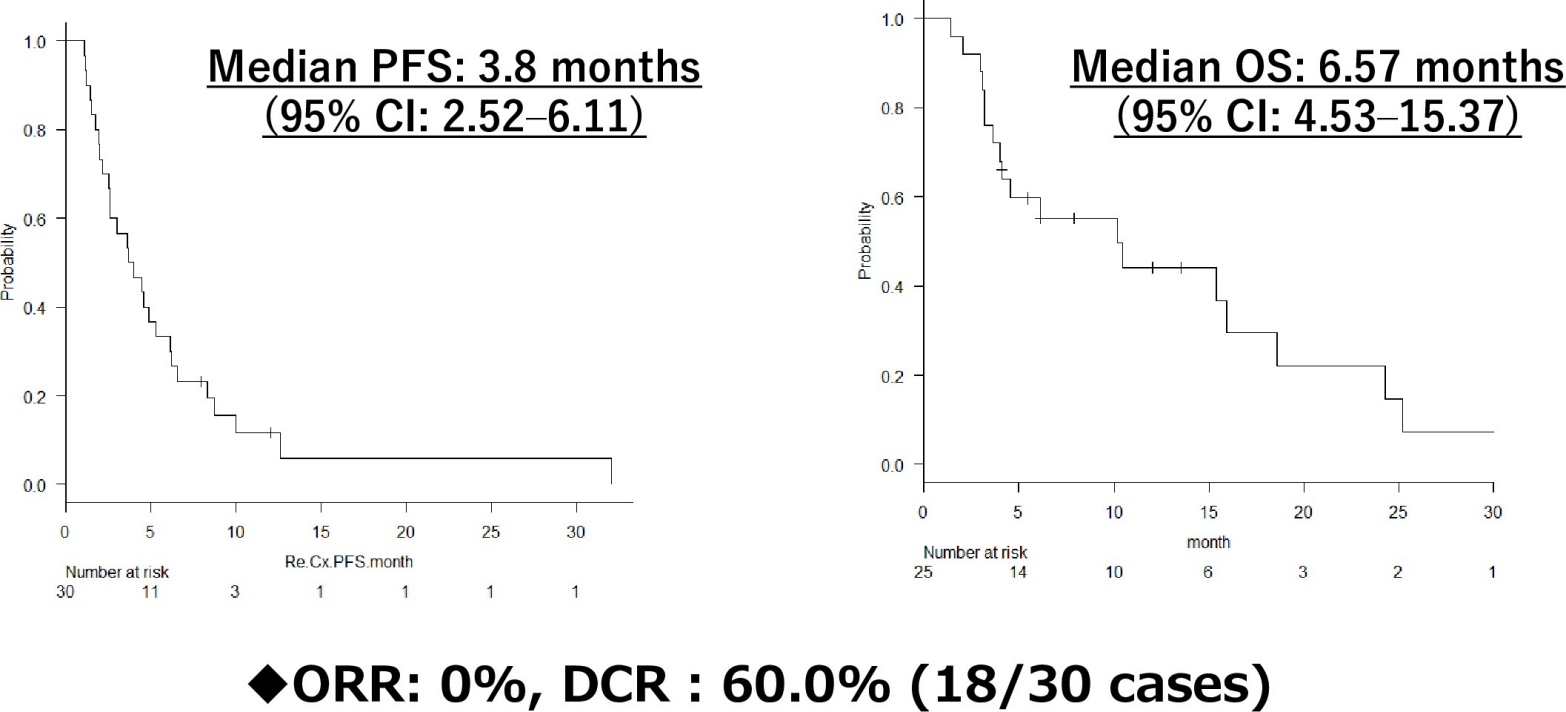
PFS, OS, ORR, and DCR of Re-Cx for 30 cases.

Following the initiation of Re-Cx, the median PFS was 3.8 months (95% CI: 2.52–6.11) and the median OS was 6.57 months (95% CI: 4.53–15.37) (Fig 1). The ORR and DCR were 0% and 60%, respectively.

### TGR

**Fig 2:**
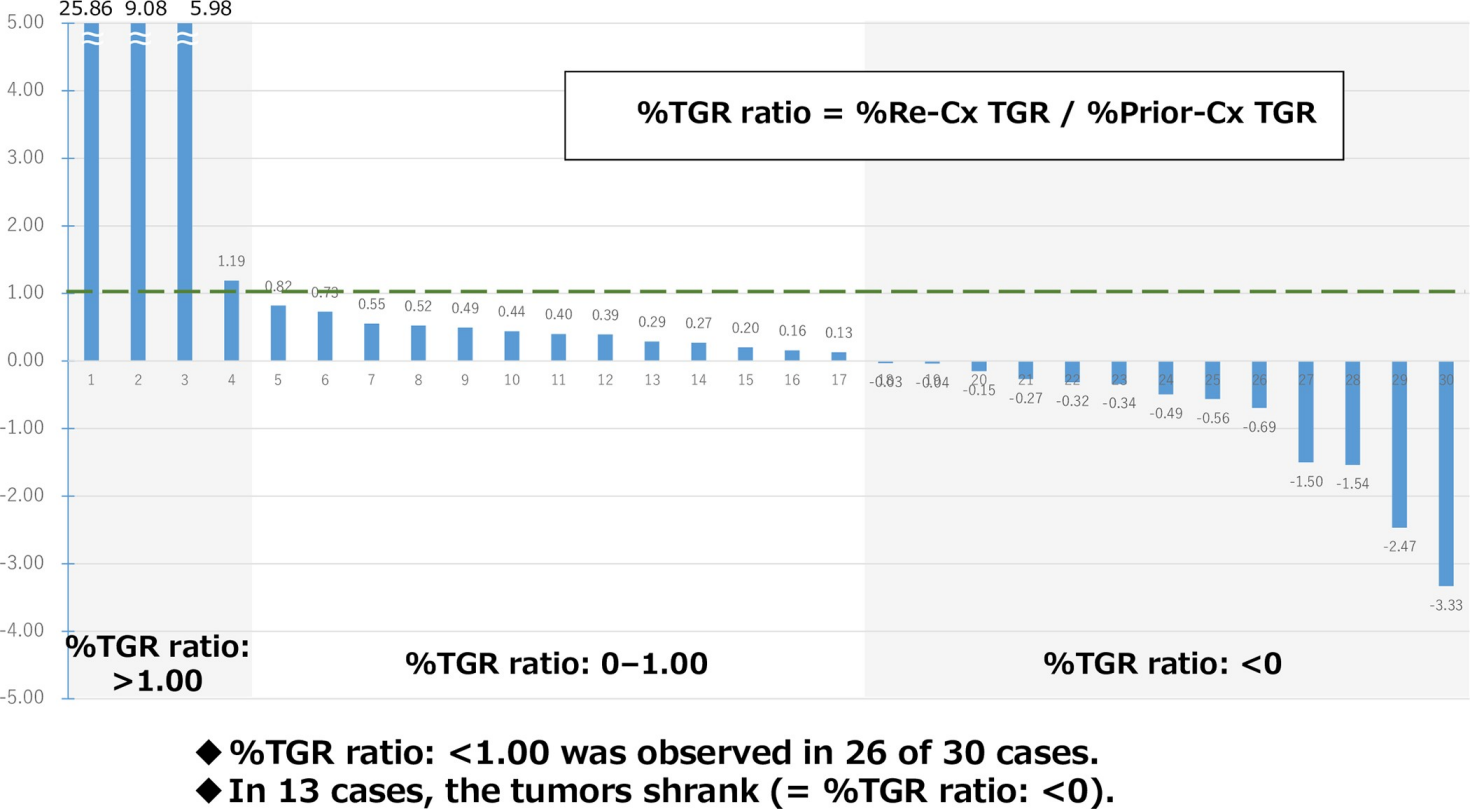
%TGR ratio in Re-Cx compared to that of Prior-Cx.

The clinical course during the initial Re-Cx is shown in Table 2. The median %TGR in Prior-Cx and Re-Cx was 7.58 (range: +0.05–+24.84), and 0.84 (range: −16.27–+16.39) (Table 3 and S1 Table). Fig 2 shows the ratio of %TGR in Re-Cx compared with that of Prior-Cx for each case. In 26 of 30 cases (86.7%), the ratio of %TGR was <1.0, indicating that the TGR after Re-Cx was slower than that observed during Prior-Cx. In 13 of 30 cases (43.3%), the ratio of %TGR was <0, representing some tumor shrinkage.

We divided the 30 cases into three groups according to the Re-Cx regimens (oxaliplatin-based, irinotecan-based, and others). The proportions of patients showing the ratio of %TGR <1.0 in these groups were 89.5% (n = 17/19), 87.5% (n = 7/8), and 66.7% (n = 2/3), respectively. Those <0 were 42.1% (n = 8/19), 62.5% (n = 5/8), and 0% (n = 0/3), respectively. Furthermore, according to the two types of molecular targeted agents used in Re-Cx (bevacizumab, and cetuximab/panitumumab), the proportions of patients with the ratio of %TGR <1.0 were 100% (n = 12/12) and 62.5% (n = 5/8), respectively. Those <0 were 50% (n = 6/12) and 37.5% (n = 3/8), respectively.

S2 Fig shows the relationship between ratio of the %TGR in the Re-Cx and duration, interval and best response in the initial Re-Cx. There were no clear relationships between the ratio of %TGR and treatment duration of the initial Re-Cx (*p* = 0.534) (S2 Fig), interval from the last administration of initial Re-Cx to the initiation of Re-Cx (*p* = 0.562) (S3 Fig), or best reduction of tumor size during the initial Re-Cx (*p* = 0.102) (S4 Fig).

## Discussion

In summary, although there was no response to Re-Cx, the treatment resulted in some tumor shrinkage (43%), slower TGR compared with that showing disease progression during Prior-Cx (87%), and was associated with DCR of 60% and a median PFS and OS of 3.8 and 6.6 months, respectively. Considering that phase III trials of regorafenib (ORR 1.0%, DCR 41%, median PFS 1.9 months, median OS 6.4 months) and TAS-102 (ORR 1.6%, DCR 44%, median PFS 2.0 months, median OS 7.1 months) [6, 7] showed comparable efficacy to that recorded in our results, it is suggested that Re-Cx may have some impact on patient outcome.

The phase II study (RE-OPEN study) reported the efficacy of reintroduction of oxaliplatin in 33 patients with mCRC. Twelve weeks after reintroduction, the response rate was 6.1% (2/33), the DCR was 39.4% (13/33), and tumor shrinkage was observed in 45.5% of patients. The median PFS and OS were 98.0 days (95% CI: 55.7–140.3) and 300.0 days (95% CI: 229.3–370.7), respectively [20]. Another retrospective study also reported the efficacy of oxaliplatin re-challenge in 110 patients with mCRC. The response rate and DCR were 30.9% (34/110) and 68.2% (75/110), respectively. The median PFS and OS were 5.9 months (95% CI: 4.4–7.4 months) and 18.5 months (95% CI: 14.0–23.0 months), respectively [21]. By comparing the results of our study with those of previous reports, the present response rate appeared to be worse (6.1% vs. 30.9% and 0%, respectively). A possible reason for the worse results observed in our study may be differences in the background of patients. Previous studies reused only oxaliplatin as a chemotherapeutic agent, and the subjects were limited to patients who had shown response during the first administration of this drug. Moreover, in these two previous reports, the initial administration of oxaliplatin was discontinued due to disease progression or toxicities, while all prior oxaliplatin was discontinued due to disease progression in this study. It is reasonable that sensitivity to oxaliplatin may remain after discontinuation due to toxicities; however, that may not be the case after disease progression.

Regarding re-challenge with irinotecan, the BOND study showed that irinotecan combined with cetuximab exhibited better efficacy than cetuximab alone in patients with colorectal cancer refractory to irinotecan [22]. Similarly, irinotecan combined with bevacizumab plus cetuximab resulted in a higher response rate than bevacizumab plus cetuximab in the same setting. It is suggested that resistance to irinotecan may be partly resolved through combination with cetuximab [23]. However, these two previous studies enrolled cetuximab-naïve patients. Notably, the five patients included in our study, who had shown disease progression during prior chemotherapy with anti-epidermal growth factor receptor (anti-EGFR) antibodies, did not show response during Re-Cx using irinotecan plus cetuximab. It is speculated that sensitivity to irinotecan could not be resumed by rechallenge with anti-EGFR antibodies which had been discontinued due to disease progression. On the other hand, a couple of studies investigated the efficacy of re-challenge using anti-EGFR antibodies [24, 25]. However, in this study, none of the eight patients who received Re-Cx containing anti-EGFR antibodies achieved response and five of those showed PD (S3 Table). The clinical significance of re-challenge with anti-EGFR antibodies is currently under investigation in a clinical trial (WJOG8916G).

Re-challenge with anti-angiogenic agents (e.g., bevacizumab, ramucirumab, and aflibercept), as second-line treatment beyond progression showed a survival benefit [26]. In our study, 12 of 30 cases received bevacizumab in the Re-Cx. Among those, the proportion of the ratio of %TGR <1.0 was 100% (n = 12/12) and nine patients (75%) showed stable disease after Re-Cx with bevacizumab. Although bevacizumab does not have the ability to independently shrink tumors, it may play a role in reducing the TGR in Re-Cx even after two or three times failures of anti-angiogenic agents. For example, bevacizumab as first line, anti-vascular endothelial growth factor drugs (e.g., bevacizumab, ramucirumab, aflibercept) as second line, and regorafenib thereafter.

Comparison showed that the TGR of Re-Cx was slower than that recorded during Prior-Cx in 26 of 30 cases (87%). The slower TGR could be considered clinical benefit for the patients after failure of standard treatment. Notably, a ratio of %TGR <0, which indicates tumor shrinkage, was obtained in 13 of 30 cases (43.3%). These results also suggest the benefit of Re-Cx.

We also investigated the factors associated with the ratio of %TGR to identify optimal candidates for Re-Cx. According to previous studies, a longer oxaliplatin-free interval and initial sensitivity to oxaliplatin with disease control were reported to be criteria for the administration of Re-Cx [27, 28], as a part of acquired resistant mechanism may disappear time-dependently if not exposed to some agents. A better PS, solitary metastasis, and disease control after 12 weeks of treatment were independent factors influencing PFS in the RE-OPEN study [17]. However, in this study, there was no association between the ratio of the %TGR after the Re-Cx and interval from the initial Re-Cx. In addition, there was no clear relationship between the ratio of %TGR and efficacy during the initial-Cx. The various Re-Cx regimens used involve numerous mechanisms of resistance. Some of these mechanisms may disappear with time, whereas others may not. Thus, it is difficult to find a certain relationship between the interval and efficacy of the Re-Cx. The median treatment lines of the initial Re-Cx and Re-Cx were first and sixth, respectively. This implies that the agent-free interval in this study was relatively long for many cases, and that a long interval may increase sensitivity to treatment and lead to favorable efficacy of the Re-Cx.

Our study had several limitations, namely its retrospective nature, the small number of patients who met the eligibility criteria that may have led to selection bias, absence of control (e.g., best supportive care), lack of information on adverse events, and unavailability of data concerning quality of life. Therefore, it cannot be concluded that Re-Cx is recommended as salvage treatment in clinical practice. And since 5 out of 25 people have Re-Cx twice, there may be some bias compared to first Re-Cx cases. However, as a result of sensitivity analysis of 25 cases excluding 5 cases of the second Re-Cx, there was no difference from 30 cases. Therefore, we think there is little bias.

In conclusion, Re-Cx may have modest efficacy against mCRC. Further investigations are warranted to determine the most appropriate subset of patients for this treatment.

## Supporting information

S1 Fig Flow diagram. (TIF)

S2 Fig %TGR ratio and treatment duration of initial Re-Cx (30 cases). (TIF)

S3 Fig %TGR ratio and interval period from the last administration of initial Re-Cx to beginning of Re-Cx (30 cases). (TIF)

S4 Fig %TGR ratio and best tumor reduction rate in initial Re-Cx (24 cases). (TIF)

S1 Table Re-Cx regimens of 30 cases classified according to the cytotoxic agents. (DOCX)

S2 Table Details of initial Re-Cx regimens and Re-Cx regimens. (DOCX)

S3 Table Re-Cx regimens (30 cases) classified according to the molecular targeted agents. (DOCX)
