# Supplementary figures and images for: Tumor growth rate during re-challenge chemotherapy with previously used agents as salvage treatment for metastatic colorectal cancer: A retrospective study
Source: PLoS One. 2021 Sep 24;16(9):e0257551. doi: 10.1371/journal.pone.0257551 (PMC8462714; doi:10.1371/journal.pone.0257551)

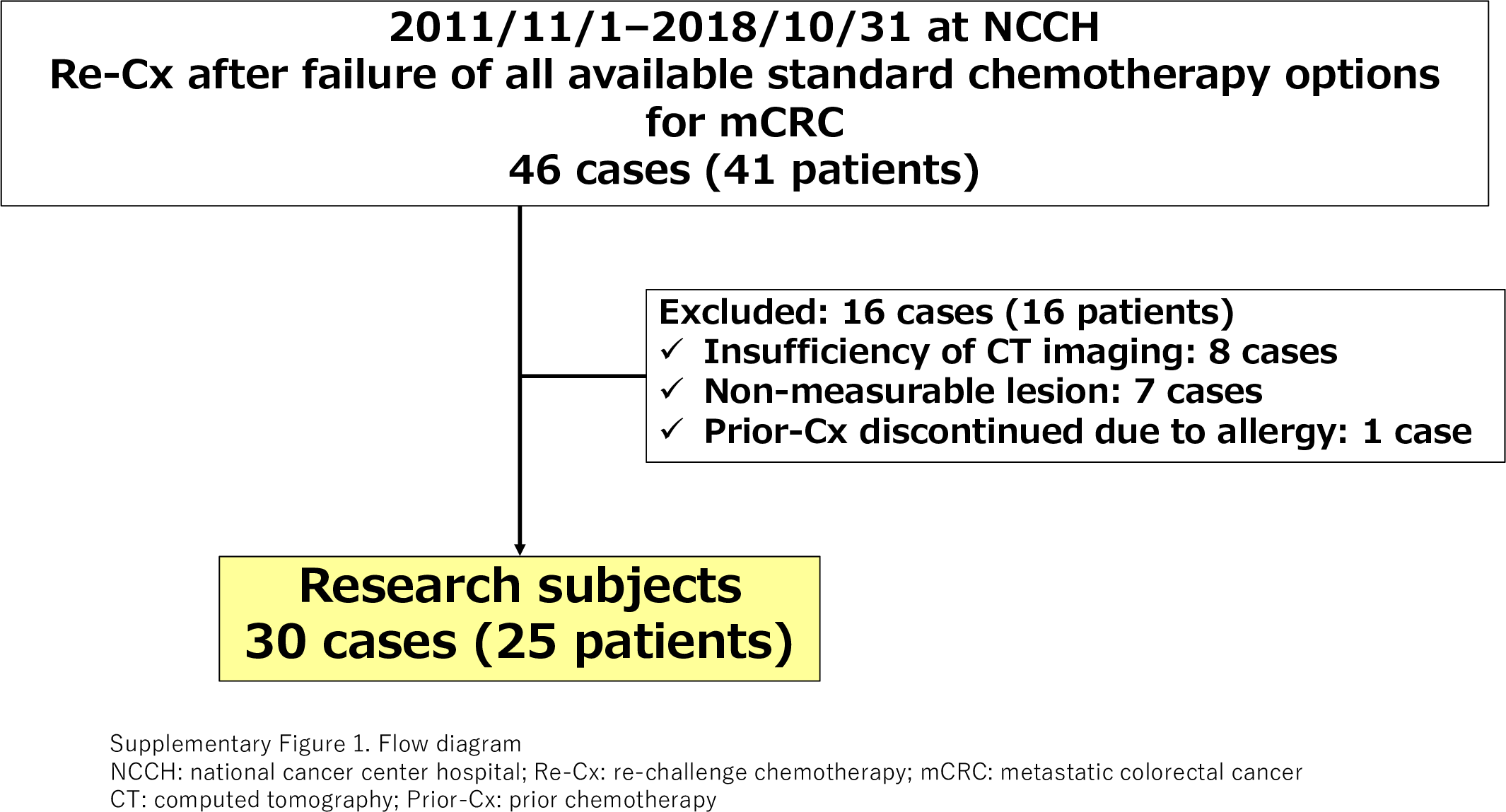

Supplement: S1 Fig — (TIF) [file pone.0257551.s001.tif]

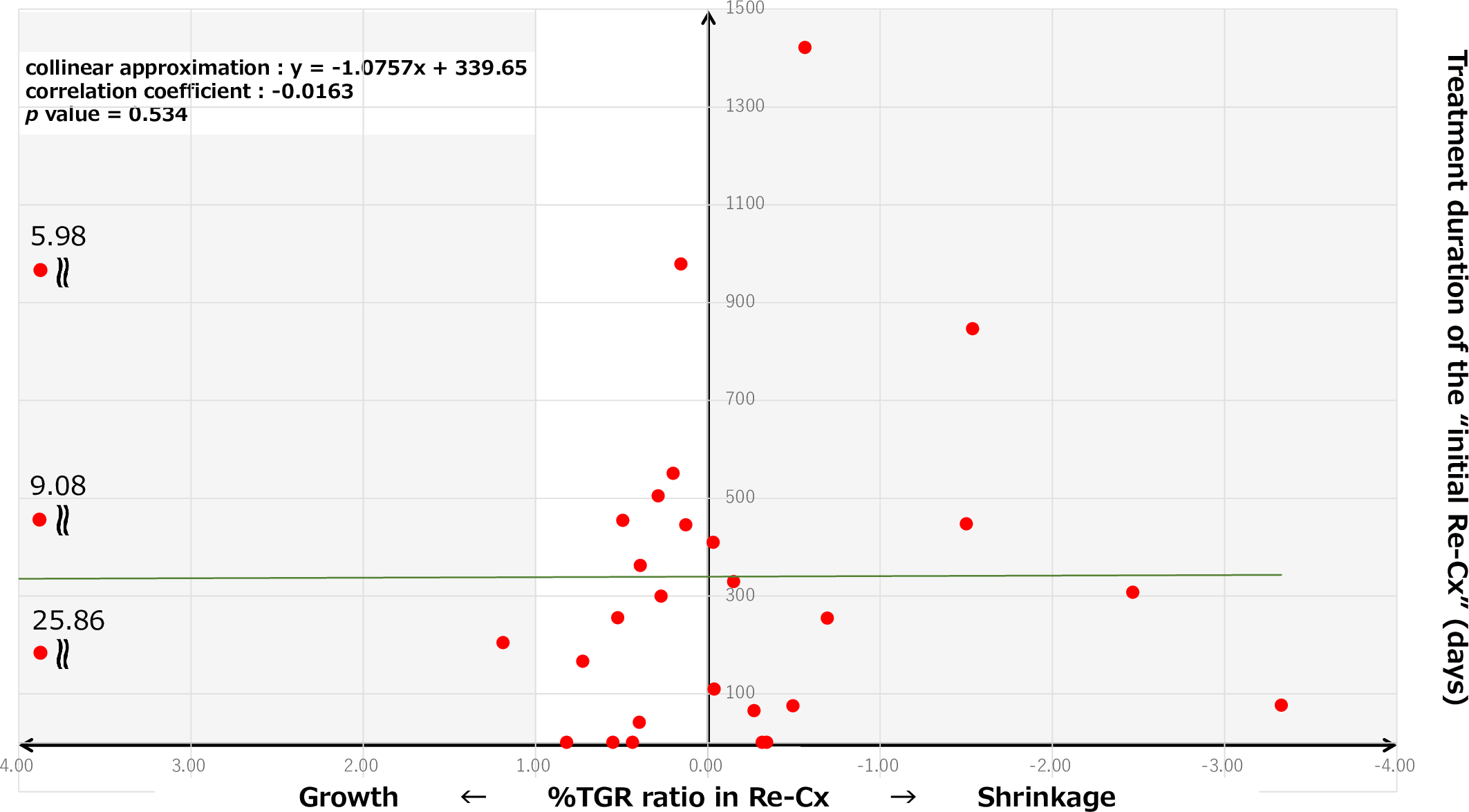

Supplement: S2 Fig — (TIF) [file pone.0257551.s002.tif]

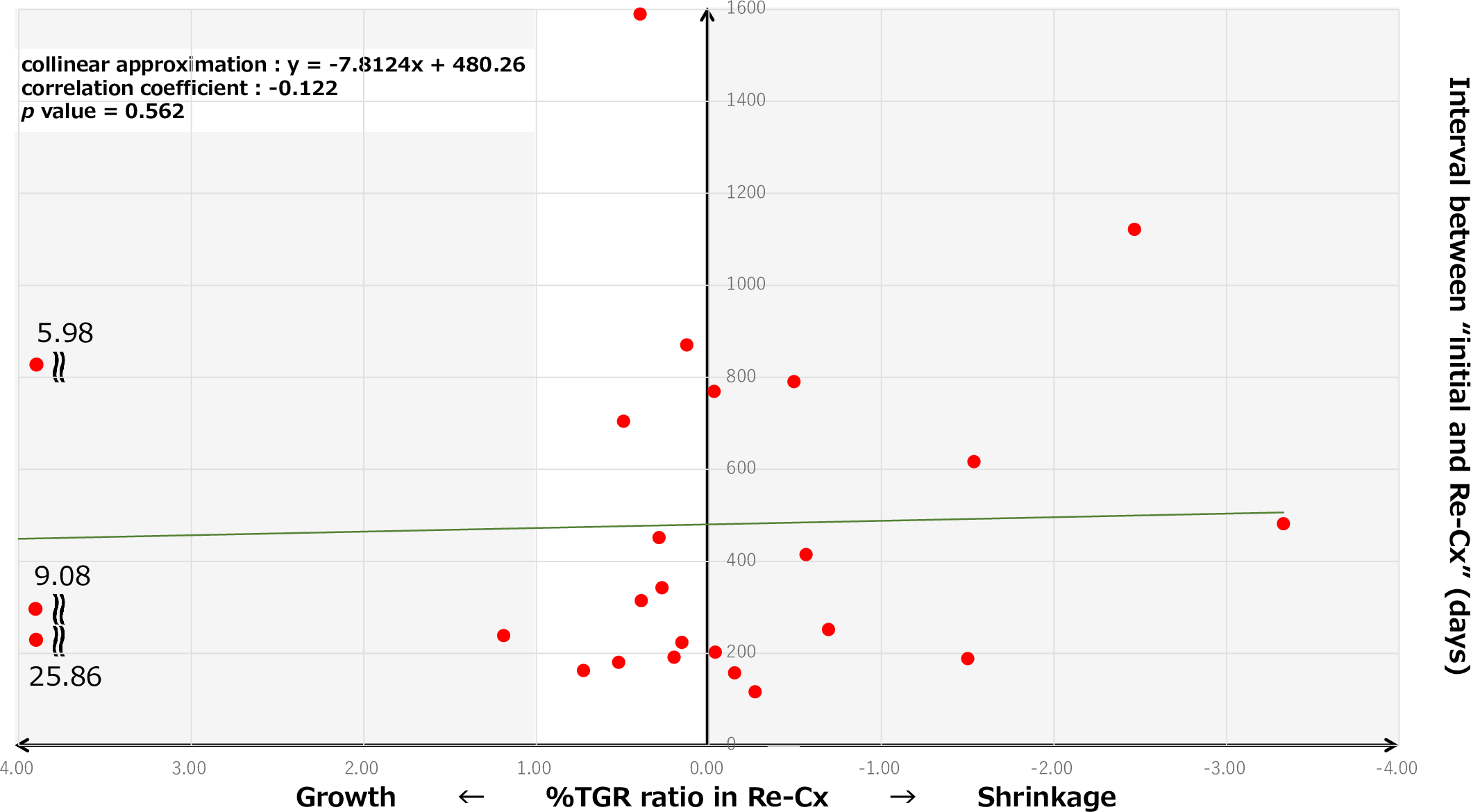

Supplement: S3 Fig — (TIF) [file pone.0257551.s003.tif]

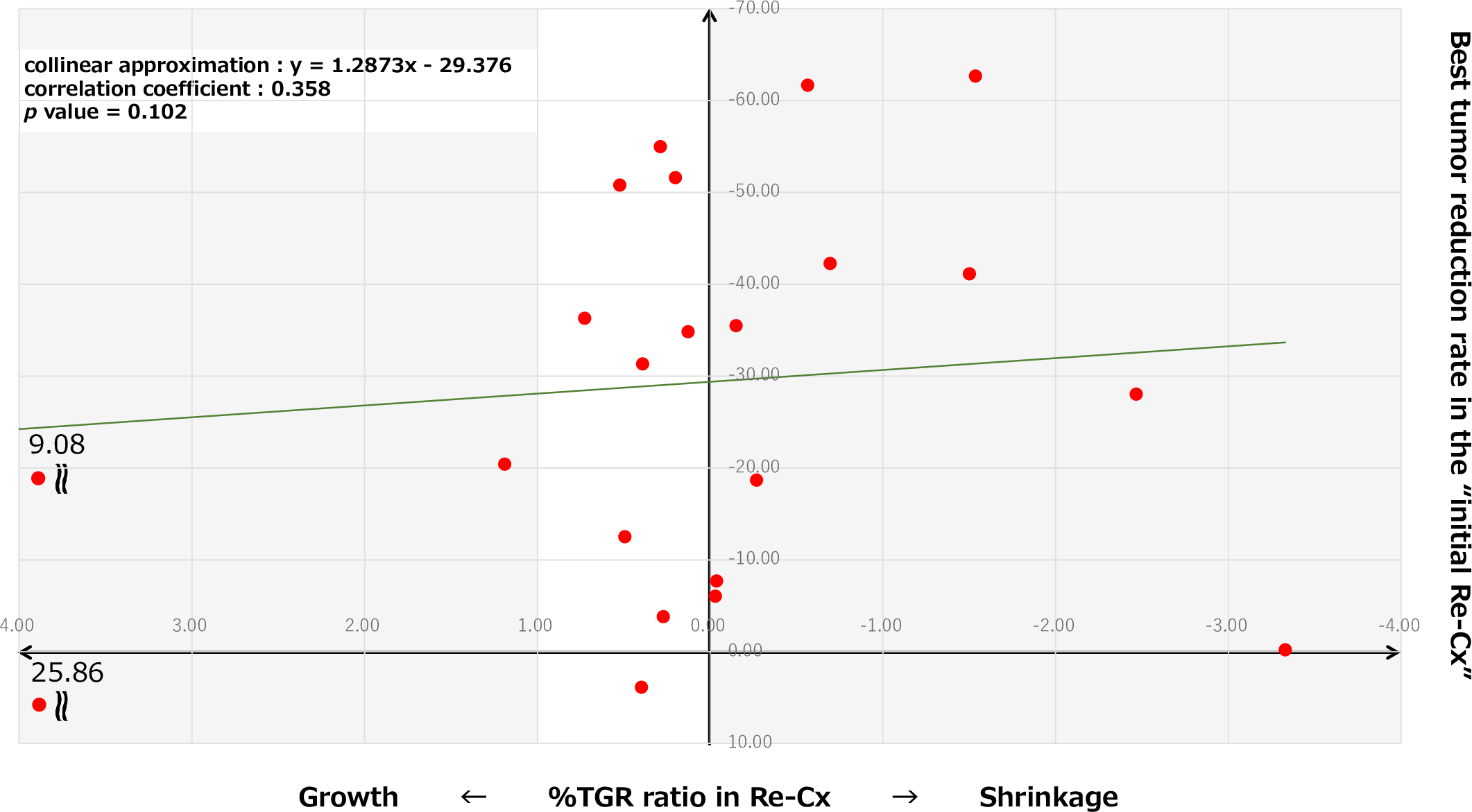

Supplement: S4 Fig — (TIF) [file pone.0257551.s004.tif]
