# Supplementary material for: Tumor growth rate during re-challenge chemotherapy with previously used agents as salvage treatment for metastatic colorectal cancer: A retrospective study
Source: PLoS One. 2021 Sep 24;16(9):e0257551. doi: 10.1371/journal.pone.0257551 (PMC8462714; doi:10.1371/journal.pone.0257551)
Supplement: S1 Table — (DOCX) [file pone.0257551.s005.docx]

S1 Table. Re-Cx regimens of 30 cases classified according to the cytotoxic agents

| Group | Regimen | Treatment line  4/5/6/>6 | Best Response  CR/PR/SD/PD | Proportion of %TGR,  median [range] | Treatment duration,  median [range] (months) |
| --- | --- | --- | --- | --- | --- |
| OHP base  (n=19) | FOLFOX  (n=7) | 3 / 0 / 3 / 1 | 0 / 0 / 3 / 4 | 1.17  [−4.39–7.54] | 1.4  [0.5–7.9] |
|  | FOLFOX+BV  (n=5) | 0 / 1 / 1 / 3 | 0 / 0 / 4 / 1 | 0.87  [−16.27–3.12] | 2.6  [2.3–9.3] |
|  | CapeOX+BV  (n=3) | 1 / 0 / 1 / 1 | 0 / 0 / 2 / 1 | −5.96  [−6.95–6.40] | 4.5  [0.7–6.9] |
|  | FOLFOX+Pmab  (n=1) | 0 / 0 / 1 / 0 | 0 / 0 / 0 / 1 | 16.39 | 1.6 |
|  | CapeOX  (n=1) | 0 / 0 / 1 / 0 | 0 / 0 / 1 / 0 | −4.73 | 7.7 |
|  | SOX  (n=1) | 0 / 0 / 0 / 1 | 0 / 0 / 1 / 0 | −7.58 | 2.2 |
|  | SOX+BV  (n=1) | 1 / 0 / 0 / 0 | 0 / 0 / 1 / 0 | 3.01 | 3.7 |
|  | Subtotal  (n=19) | 6 (4–11) | 0 / 0 / 12 / 7 | 0.87  [−16.27–16.39] | 2.4  [0.5–9.3] |
| CPT-11 base  (n=8) | CPT-11+Cmab  (n=4) | 1 / 1 / 1 / 1 | 0 / 0 / 2 / 2 | 0.30  [−8.73–3.06] | 3.2  [1.4–6.6] |
|  | CPT-11+Pmab  (n=1) | 0 / 0 / 1 / 0 | 0 / 0 / 1 / 0 | −12.00 | 4.1 |
|  | FOLFIRI+BV  (n=2) | 0 / 0 / 0 / 2 | 0 / 0 / 1 / 1 | 2.02  [−0.16–4.22] | 2.9  [1.0–4.7] |
|  | CPT-11+BV  (n=1) | 0 / 1 / 0 / 0 | 0 / 0 / 1 / 0 | −3.94 | 5.6 |
|  | Subtotal  (n=8) | 6 (4–9) | 0 / 0 / 5 / 3 | −0.44  [−12.00–4.22] | 3.9  [1.0–6.6] |
| Others  (n=3) | Cmab  (n=1) | 0 / 0 / 0 / 1 | 0 / 0 / 0 / 1 | 5.02 | 1.2 |
|  | TAS-102+Pmab (n=1) | 0 / 0 / 0 / 1 | 0 / 0 / 0 / 1 | 8.10 | 1.2 |
|  | S-1  (n=1) | 0 / 1 / 0 / 0 | 0 / 0 / 1 / 0 | 0.51 | 3.3 |
|  | Subtotal  (n=3) | 8 (5–8) | 0 / 0 / 1 / 2 | 5.02  [0.51–8.10] | 1.2  [1.2–3.3] |
| Total |  | 6 (4–11) | 0 / 0 / 18 / 12 | 0.84  [−16.27–16.39] | 2.6  [0.5–9.3] |
