# Supplementary material for: Tumor growth rate during re-challenge chemotherapy with previously used agents as salvage treatment for metastatic colorectal cancer: A retrospective study
Source: PLoS One. 2021 Sep 24;16(9):e0257551. doi: 10.1371/journal.pone.0257551 (PMC8462714; doi:10.1371/journal.pone.0257551)
Supplement: S2 Table — (DOCX) [file pone.0257551.s006.docx]

S2 Table. Details of initial Re-Cx regimens and Re-Cx regimens

| Case | Initial Re-Cx regimens | | Re-Cx regimens | | |
| --- | --- | --- | --- | --- | --- |
|  | Regimen | Treatment line | Groups | Regimen | Treatment line |
| 1 | CapeOX | 1 | **OHP base**  **(n=19)** | FOLFOX | 4 |
| 2 | FOLFOX+BV | 1 |  | FOLFOX | 4 |
| 3 | CapeOX+BV | 2 |  | FOLFOX | 4 |
| 4 | FOLFOX | 1 |  | FOLFOX | 6 |
| 5 | FOLFOX+BV | 1 |  | FOLFOX | 6 |
| 6 | SOX+BV | 2 |  | FOLFOX | 6 |
| 7^(1)^ | FOLFOX+BV | 1 |  | FOLFOX | 11 |
| 8^(2)^ | FOLFOX+BV | 1 |  | FOLFOX+BV | 5 |
| 9 | FOLFOX+BV | 1 |  | FOLFOX+BV | 6 |
| 10 | SOX+BV | 2 |  | FOLFOX+BV | 7 |
| 11 | FOLFOX+BV | 6 |  | FOLFOX+BV | 8 |
| 12^(3)^ | FOLFOX+BV | 1 |  | FOLFOX+BV | 9 |
| 13 | CapeOX+BV | 1 |  | FOLFOX+Pmab | 6 |
| 14 | FOLFOX | 1 |  | CapeOX | 6 |
| 15 | CapeOX+BV | 1 |  | CapeOX+BV | 4 |
| 16 | SOX+BV | 1 |  | CapeOX+BV | 6 |
| 17^(4)^ | SOX+BV | 2 |  | CapeOX+BV | 9 |
| 18^(5)^ | FOLFOX+BV | 2 |  | SOX | 7 |
| 19 | CapeOX+BV | 1 |  | SOX+BV | 4 |
| 20 | FOLFIRI+Pmab | 1 | **CPT-11 base**  **(n=8)** | CPT-11+Cmab | 4 |
| 21^(4)^ | CPT-11+Cmab | 3 |  | CPT-11+Cmab | 5 |
| 22 | FOLFIRI+Cmab | 2 |  | CPT-11+Cmab | 6 |
| 23^(1)^ | CPT-11+Cmab | 5 |  | CPT-11+Cmab | 7 |
| 24 | FOLFIRI+Pmab | 1 |  | CPT-11+Pmab | 6 |
| 25^(5)^ | FOLFIRI+BV | 1 |  | CPT-11+BV | 5 |
| 26 | FOLFIRI+BV | 2 |  | FOLFIRI+BV | 7 |
| 27^(2)^ | FOLFIRI+BV | 3 |  | FOLFIRI+BV | 8 |
| 28^(3)^ | FOLFIRI+BV | 2 | **Others**  **(n=3)** | S-1 | 5 |
| 29 | CPT-11+Cmab | 4 |  | Cmab | 8 |
| 30^(6)^ | TAS-102 | 4 |  | TAS-102+Pmab | 8 |

(1, 2, 3, 4, 5): Patients received two Re-Cx regimes.

(6): Patient also received Pmab at 3 line previously.
